# Fabrication of Injectable Kartogenin-Conjugated Composite Hydrogel with a Sustained Drug Release for Cartilage Repair

**DOI:** 10.3390/pharmaceutics15071949

**Published:** 2023-07-14

**Authors:** Chao Li, Yubo Liu, Tujun Weng, Muyuan Yang, Xing Wang, Wei Chai

**Affiliations:** 1 Senior Department of Orthopedics, The Fourth Medical Center of PLA General Hospital, Beijing 100048, China; 2 National Clinical Research Center for Orthopedics, Sports Medicine & Rehabilitation, Beijing 100853, China; 3 Beijing National Laboratory for Molecular Sciences, Institute of Chemistry, Chinese Academy of Sciences, Beijing 100190, China

**Keywords:** cartilage tissue regeneration, kartogenin, hydrogel scaffold, Schiff base, injectability

## Abstract

Cartilage tissue engineering has attracted great attention in defect repair and regeneration. The utilization of bioactive scaffolds to effectively regulate the phenotype and proliferation of chondrocytes has become an elemental means for cartilage tissue regeneration. On account of the simultaneous requirement of mechanical and biological performances for tissue-engineered scaffolds, in this work we prepared a naturally derived hydrogel composed of a bioactive kartogenin (KGN)-linked chitosan (CS-KGN) and an aldehyde-modified oxidized alginate (OSA) via the highly efficient Schiff base reaction and multifarious physical interactions in mild conditions. On the basis of the rigid backbones and excellent biocompatibility of these two natural polysaccharides, the composite hydrogel demonstrated favorable morphology, easy injectability, good mechanical strength and tissue adhesiveness, low swelling ratio, long-term sustainable KGN release, and facilitated bone marrow mesenchymal stem cell activity, which could simultaneously provide the mechanical and biological supports to promote chondrogenic differentiation and repair the articular cartilage defects. Therefore, we believe this work can offer a designable consideration and potential alternative candidate for cartilage and other soft tissue implants.

## 1. Introduction

Articular cartilage is inherently free of blood vessels and has a low cell count. Once cartilage is damaged, it cannot repair itself, which significantly limits its self-recurrence and leads to progressive degeneration of the entire joint, resulting in severe osteoarthritis. In order to prevent further deterioration of the invasive lesions of articular cartilage, several clinical managements have been well developed using surgical interventional treatments, such as osteochondral transplantation, autologous chondrocyte implantation, bone marrow stimulation, and others [1,2,3,4]. However, current approaches in recent years are considered controversial because they possess a variety of inherent drawbacks, such as donor site morbidity, potential immunogen rejection, and insufficient transplantation resources [5,6,7]. Therefore, the advent of tissue engineering, based on the three main elements of scaffolds, growth factors, and seed cells, represents a new dawn and innovative therapeutic modality for cartilage tissue regeneration.

Along with the rapid development of tissue engineering technology, the scaffold material is gradually regarded as the most important factor in tissue engineering for repairing defective tissues [8,9,10,11,12]. For example, hydrogels can simulate the compositions, structures, and properties of native cartilage, so as to promote the growth, proliferation, and differentiation of cells in the defect site, thus facilitating tissue regeneration [13,14,15,16,17,18]. Naturally derived polysaccharide hydrogels with porous networks, suitable mechanics, and designable biological properties have made great progress for several decades and proved to be crucial ingredients in cell growth, adhesion, proliferation, and differentiation [19,20,21,22,23]. Among them, chitosan (CS), as a sole alkaline polysaccharide in nature, has excellent biocompatibility, tailored biodegradation, and beneficial chondrocyte phenotype expression for wide bio-application, but its poor solubility, weak strength, and difficult processability are still cumbersome problems that need to be solved. Therefore, it is necessary to introduce other polymeric moieties into CS-based systems to enhance chitosan’s properties [24,25,26,27,28]. Consequently, sodium alginate (SA) and its derivative of oxidized sodium alginate (OSA) are frequently incorporated into CS-based systems due to their good accessibility and non-immunogenicity [29,30]. OSA can be easily chemically linked with the amine groups of CS polymers via the Schiff base bonds to yield composite hydrogels in mild conditions. Moreover, its carboxyl and hydroxyl groups within the polymeric chains can further allow the OSA to crosslink with the CS backbone through hydrogen interactions and multiple ionic interactions to increase crosslinking density, enhance mechanical properties, and decrease water absorption.

Numerous studies have shown that kartogenin (KGN), as an excellent chondrogenic promoter, can maintain biological activity and maintain good stability, thus promoting long-term chondrogenesis in cartilage tissue repair. After screening 22,000 structurally diverse and heterocyclic drug-like small molecules, it was found that KGN, without any toxicity, could obviously improve the chondrocyte differentiation of MSCs in a dose-dependent manner [31]. Moreover, KGN also has longer stability and half-life than those of the biological protein growth factors in the application of cartilage tissue engineering [32,33,34,35,36]. In addition, the lymphatic system can rapidly clear the pure KGN injected via the articular cavity, and this intra-articular injection can also cause organ damage due to leakage into the circulatory system. Thus, a drug delivery system for the controlled KGN release is urgently required and needed [33,37,38]. However, most of the KGN-based drug delivery methods relied on physical encapsulation, which caused the initial burst release, uncontrolled drug delivery, and later difficulty in long-term release [39]. On the basis of the above background information, the pursuit of simultaneously satisfied mechanical and chondrogenic properties of hydrogel scaffolds as well as a novel delivery system for bioactive KGN is a topic worthy of deep investigation and clinical exploitation in order to enhance the therapeutic effect of cartilage repair.

**Figure 1:**
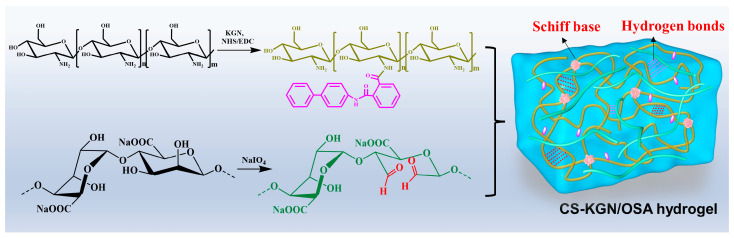
Schematic illustrations of synthesis pathways of CS-KGN and OSA polysaccharides and the CS-KGN/OSA composite hydrogel.

It is difficult for cartilage scaffolds to simultaneously meet the requirements of biocompatibility, degradability, mechanics, and microenvironment supplies, thus significantly limiting their widespread use. In this work, combining the advantages of the efficient Schiff base reaction between the polysaccharide KGN-conjugated chitosan (CS-KGN) and OSA polymer (Figure 1), we tried to prepare a kind of composite hydrogel with favorable mechanical and biological properties and investigated its application in cartilage tissue engineering. Relying on the chemical modification methods, the bioactive KGN molecules were chemically linked into the CS backbone while the OSA macromolecule was obtained in the presence of antioxidant NaIO_4_. Since the residual aldehyde groups within the OSA could further be reacted with the amino groups of tissue protein, the Schiff base reaction could allow the injectable hydrogels to fill up almost all areas of cartilage defect. In addition, they exhibited good biocompatibility that, in combination with the ECM, facilitated cell survival, adhesion, growth, and proliferation. Moreover, in view of the local delivery of KGN molecules along with the polysaccharide degradation, the optimized CS-KGN/OSA hydrogel was able to locally deliver the stable chondrogenesis KGN promoter, and support cell growth and chondrogenic differentiation of the encapsulated BMSCs, thus effectively maintaining the phenotype and function and elucidating the therapeutic effect on cartilage repair.

Therefore, this study aimed to prepare a biocompatible hydrogel with a low swelling ratio and favorable mechanical and long-term sustainable KGN release capacities. Even without loaded chondrotropic drugs, a CS/OSA hydrogel scaffold could facilitate the cell adhesion, growth, proliferation, and chondrogenic differentiation of BMSCs, exhibiting a suitable cell-supporting effect for potential tissue engineering. In addition, KGN is an excellent chondrogenesis promoter that induces mesenchymal stem cells to homing to promote long-term chondrogenesis in the process of cartilage tissue repair. Compared with the initial burst release and uncontrolled drug delivery of physical KGN encapsulation, we are also aiming to fabricate the chemically linked CS-KGN polymer and corresponding CS-KGN/OSA hydrogel with stable and long KGN release in situ to collaboratively promote chondrogenesis. Consequently, compared with the previous attempts to construct engineered scaffolds in order to achieve tissue regeneration, the current trial presents the synergistic effects of a hydrogel and its potential to provide a clinical alternative that highlights excellent biocompatibility, ease of injection, flexibility of use, and long-lasting chondrogenesis.

## 2. Materials and Methods

### 2.1. Materials

Chitosan (CS, degree of deacetylation > 90%, viscosity 45 mPas, Mw ≈ 10 kPa, Shandong Jinhu Co., Ltd., Zibo, China), sodium alginate (SA, 98%, Energy Chemical), kartogenin (KGN, 95%, Selleckchem Co., Ltd., Houston, TX, USA), N-hydroxysuccinimide (NHS, 99%, Energy Chemical), 1-ethyl-3-(dimethylaminopropyl) carbodiimide hydrochloride (EDCI, 99%, Energy Chemical), proteinase K (Solarbio Science & Technology Co., Ltd., Beijing, China), sodium periodate (NaIO_4_, J&K), and fibrin glue (Guangzhou Bioseal Biotech, Co., Ltd., Guangzhou, China) were used. All the other biochemical reagents were directly purchased from Sigma-Aldrich (St. Louise, MO, USA) and used without any further treatment steps.

### 2.2. Synthesis of the CS-KGN Polymer

Briefly, an appropriate molar of KGN (2 mmol, 0.64 g), NHS (2.4 mmol, 0.28 g), and EDCI (2.4 mmol, 0.46 g) were mixed in the DMSO/H_2_O solutions under vigorous stirring. After mixing for 4 h at room temperature, 5 wt% of CS (0.36 g) solution was added to the mixture with vigorous stirring for another 24 h. Then, the KGN-conjugated CS was dialyzed against deionized water (MW cutoff, 2 kDa) for 2 d to remove the impurities/DMSO solvent and lyophilized to yield a purified product referred to as the CS-KGN polymer.

### 2.3. Synthesis of the OSA Polymer

Briefly, 10 g of SA was first dissolved in 200 mL of aqueous solution followed by the addition of 50 mL of anhydrous ethanol under vigorous stirring for 2 h. Then, the oxidant NaIO_4_ agent was incorporated into the solutions under the N_2_ atmosphere. After vigorous stirring overnight, 2 mL of ethylene glycol was added to reduce the remnant oxidizing agent, and then the targeted OSA polymer was precipitated after adding the anhydrous ethanol and NaCl solutions. Furthermore, the impurities were removed via the dialysis procedure (MW cutoff, 2 kDa) against deionized water for 2 d and lyophilized to yield a purified product referred to as the OSA polymer.

### 2.4. Preparation of the CS-KGN/OSA Composite Hydrogel

The CS-KGN and OSA solutions were first prepared in water at room temperature. Without adding any other additives, the CS-KGN/OSA composite hydrogel was simply prepared by adding 5 wt% of CS-KGN solution into the same equivalent volume of OSA solution (3 wt%) via the vortexing method and then stored at room temperature to allow gelation to occur. As a control, the CS/OSA hydrogel was also prepared with the same method.

### 2.5. Structural Characterizations

^1^H NMR was applied for ensuring the chemical structures of CS-KGN and OSA polymers on a Bruker DRX-400 using D_2_O. Fourier transform infrared spectroscopy (FTIR) was recorded on a TENSOR-27 spectrometer in the range 400–4000 cm^−1^ to assess the chemical linkage of CS-KGN polymer. Scanning electron microscopy (SEM) images were obtained on a JSM-6700F microscope to observe the network morphology and inner structure of CS-KGN/OSA hydrogel.

### 2.6. Compressive Strength Measurement

The compressive profile of CS-KGN/OSA hydrogel was measured using a Instron 3365 testing machine. Wherein, the cylindrical sample (diameter: 15 mm; height: 5.5 mm) was constructed with an experiment compressive speed of 1 mm/min.

### 2.7. Rheology Study

The rheological measurement was carried out using a rheometer. The CS-KGN/OSA hydrogel was spread on a 25 mm parallel plate and sealed with silicone oil to prevent solvent evaporation. The dynamic frequency scan range: 0.1–100 rad s^−1^; stress amplitude: 0.1%; and temperature: 25 °C.

### 2.8. Adhesive Study

The adhesion measurement was performed using the lap shear test by injecting 1 mL of CS-KGN/OSA onto the porcine skins, and commercially available fibrin glue was used as a control. Briefly, standard lap shear and peeling tests were performed on porcine skins that were adhered using the CS-KGN/OSA or fibrin glue for pressing 30 s before the tests, respectively. The porcine skins were cut into slices with a length of 60 mm, thickness of 10 mm, and width of 20 mm for usage. All tests were performed using a Instron 3365 testing machine under a constant rate of 10 mm min^−1^.

### 2.9. Burst Pressure Test

The burst pressure measurement was conducted using a published method [40,41]. Briefly, a segment of porcine aorta vein was first cut and then cleaned to remove the excess fat. The vein was filled with pH 7.4 PBS solutions and linked to a syringe pump. A 2 mm incision was made on the vein surface. Then, 500 µL of CS-KGN/OSA was injected into the incision. The hydrogel thickness was set at ca. 5 mm and the burst pressure was assessed after 2 min of gel formation. The peak pressure before pressure loss is considered to be the burst pressure. All the measurements were repeated three times at room temperature. Fibrin glue was used with the same parameters and conditions.

### 2.10. Swelling Behavior

Briefly, the CS-KGN/OSA hydrogel with the initial weight (W_i_) was immersed into 25 mL of pH 7.4 PBS solutions and successively weighed (W_s_) after incubation for certain periods of time (0.5, 1, 2, 4, 8, 12, 16, 24, 48, and 72 h) at 37 °C. The swelling ratio was calculated according to the equation below:

Swelling ratio (%) = (W_s_ − W_i_)/W_i_ × 100%

### 2.11. Cumulative Release of KGN In Vitro

The CS-KGN/OSA hydrogel was prepared in a container (diameter: 15 mm; height: 7.5 mm), which was then immersed into the PBS solution (pH 7.4, 3 mL) with and without proteinase K (6 U/mL) at 37 °C. After incubation and collection at the predetermined intervals, the PBS solution containing the released KGN was withdrawn and added to the same PBS solutions to keep the total volume constant, which was quantified using the standard curve to obtain the KGN release behavior using a UV−vis spectrophotometer. The absorption peak of KGN was at 279 nm and the calculation equation of cumulative release equation was used as shown below:

Cumulative release rate (%) = (released KGN/total KGN content in hydrogel) × 100%

### 2.12. Cell Seeding and Cell–Hydrogel Composite Culture

Bone marrow mesenchymal stem cells (BMSCs) were isolated from 3-month-old New Zealand white rabbits, which were reviewed and approved by the institutional Animal Care and Use Committee of PLA General Hospital (ethics approval number: KYLL20210617). Isolation, culture, trilineage differentiation potential assay, and immunophenotypic identification of BMSCs were verified as previously described in the literature [42]. Injection seeding was used to seed the MSCs to hydrogel scaffolds [12]. Cell suspension injection was carried out by injecting 60 μL of concentrated cell solution (1 × 10^5^) into the top/bottom/side of the hydrogel using a 25-gauge needle. The cells seeded on the hydrogel scaffold were the third-passage BMSCs.

For chondrogenic differentiation, the cell–hydrogel composites were incubated for 2 h to facilitate cell adhesion, and then the fresh chondrogenic differentiation medium (Cyagen Biosciences Inc., Beijing, China) was added to support further culture and the following experiments.

### 2.13. Cell Cytotoxicity and Proliferation

A Cell Counting Kit-8 assay (CCK-8, Dojindo Laboratories, Tokyo, Japan) was applied to assess the cytotoxicity of the hydrogel. The injection seeding method was used to seed the BMSCs to the hydrogels. Briefly, the cell suspension injection was performed by injecting 80 μL of concentrated cell solution (1 × 10^5^ cells) into the hydrogel using a 25-gauge needle. After incubation for 1, 2, 3, 5, and 7 days, we removed the original culture medium and replaced it with fresh culture medium (100 μL) containing 10 µL of CCK-8. The cell viability was measured with the absorbance of 450 nm on a microplate reader.

### 2.14. Live and Dead Assay

The live and dead assay method was used to visualize the survival of BMSCs on the CS-KGN/OSA composite hydrogel. After culturing for 1 d, the cell–hydrogel composite was washed with PBS to remove the culture medium and then immersed in 2 mM of calcein AM and 4 mM of ethidium homodimer-1 reagents for 1 h at 37 °C. Confocal microscopy was then used to observe the live (green) and dead (red) cells with excitation wavelengths of 568 nm and 488 nm.

### 2.15. Semiquantitative RT-PCR

After incubation for 7 and 14 days, the cell–hydrogel composite was removed from the culture medium and washed with pH 7.4 PBS solutions. After that, it was placed in a mortar and ground into a powder with liquid nitrogen. The powder was added to TRIZOL reagent (Invitrogen, Carlsbad, CA, USA) to fully lysate the cells. The total RNA was isolated by the acid guanidinium thiocyanate–phenol–chloroform extraction technique [43]. The RevertAid First Strand cDNA Synthesis Kit (K1622, Thermo Scientific, Carlsbad, CA, USA) was used to reverse-transcribe isolated RNA. According to the previous literature [12,32], real-time polymerase chain reaction (RT-PCR) analysis was performed to detect the expression of cartilage-specific marker genes using the SYBR Green PCR Master Mix Real-time PCR system. The relative expression of the target genes was calculated using the ^ΔΔ^CT method. The sequences of the primers are listed in Table 1.

### 2.16. Quantification of DNA, GAG, and COL-2 Content

Briefly, the COL2 content was tested using an ELISA kit (Cloud-Clone, Corp., Houston, TX, USA) according to the manufacturer’s instructions (Jianglai bio, JL22853) and previous literature [32]. A high-efficiency RIPA tissue/cell lysis solution (R0010, Solarbio Science & Technology Co., Ltd., Beijing, China) was used to obtain the total proteins of the cell–hydrogel composites. The content of proteoglycan was detected from the GAG content by a 1,9-dimethyl methylene blue (DMMB; Sigma, St. Louis, MO, USA) dye-binding assay. Total GAG was normalized to total DNA content. Thereafter, 10 μL of the sample was added to 100 μL of DMMB and mixed for the measurement with an absorbance of 525 nm. A standard curve was established from the chondroitin-6-sulfate derived from shark cartilages (Sigma, St. Louis, MO, USA). Hoechst33258 staining and fluorometric assay were performed to measure the DNA content of the cell–hydrogel composites. After culturing for 7 and 14 days, the cell–hydrogel composites were weighed and then digested in a prepared papain solution (Sigma, St. Louis, MO, USA) containing EDTA (0.5 M), cysteine-HCl (0.05 M), and papain enzyme (1 mg/mL) at 60 °C for 48 h to obtain aliquots of the sample digestion. A 10 μL aliquot of sample digestion was mixed with 100 μL of Hoechst33258 working solution (2 μg/mL) and incubated at 37 °C for 20 min. The fluorescence intensities were then determined using a microplate reader (Thermo, Waltham, MA, USA) at an excitation wavelength of 360 nm and an emission wavelength of 460 nm. The DNA content was normalized with a standard curve of calf thymus DNA (Sigma, St Louis, MO, USA).

### 2.17. Statistics Analysis

All results were obtained as mean ± standard deviation for more than 3 times. After the homogeneity test of variance, one-way analysis of variance (ANOVA) was used to calculate the differences between the groups, and *p* < 0.05 was considered statistically significant.

## 3. Results and Discussion

### 3.1. Preparation and Characterization of Polymers

**Figure 2:**
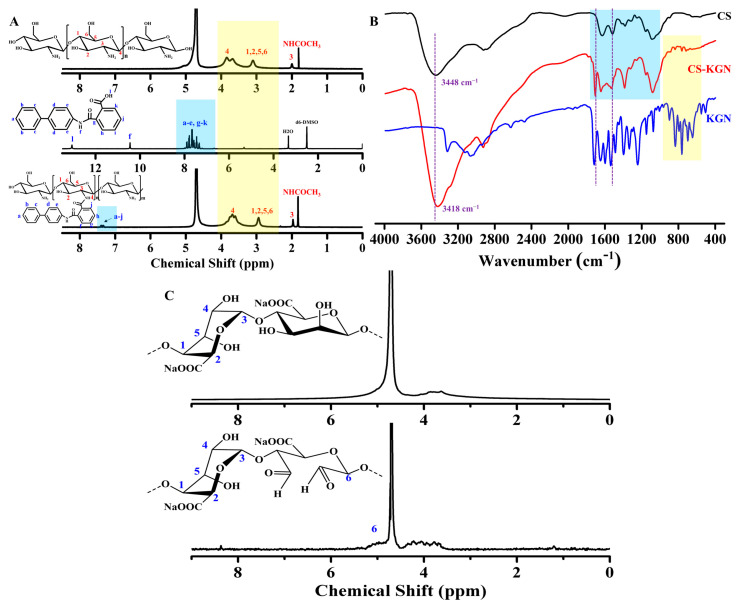
(**A**) ^1^H NMR and (**B**) IR spectra of CS, KGN, and CS-KGN polymers. (**C**) ^1^H NMR spectra of SA and OSA polymers.

The synthetic routes of CS-KGN, OSA polysaccharides, and the CS-KGN/OSA hydrogel are shown in Figure 1. KGN, as a non-protein chondrogenesis inducing agent, was testified to have abilities on the facilitation of biological activity and promotion of chondrocyte differentiation for cartilage repair. By means of the efficient amidation between the EDC/NHS-activated ester of the KGN agent and the CS in the DMSO/H_2_O solutions, the CS-KGN conjugate was simply prepared with a grafting ratio of ca. 92%. The ^1^H NMR spectrum in Figure 2A shows that the major signals of benzene groups (a–j) at δ = 7.3–7.9 ppm are attributed to the KGN moieties and the resonance peaks at δ = 1.9 and 2.7 ppm belong to the methyl (-CH_3_) and methylene proton at the C3 position of the CS polymer. Especially, these obvious shift peaks in the benzene groups and the glycoside units in Figure 2A (blue and yellow boxes) revealed the formation of amide linkages and successful preparation of the CS-KGN conjugate without the mixture of two polymers. In addition, the chemically grafting ratio of KGN onto the CS-KGN was ca. 91.5% via the ^1^H NMR spectrum in Figure 2A, indicating its highly effective amidation reaction in mild conditions. Furthermore, FT-IR spectra also provided powerful evidence for the formation of the CS-KGN conjugate. As shown in Figure 2B (blue and yellow boxes), the characteristic absorption peaks of vibration (C-N) at ca. 3418 cm^−1^ and amide I and II bands at ca. 1640 cm^−1^ and 1545 cm^−1^ were basically attributed to the deacetylated CS polymer and CS-KGN polymer, but the explicit signal shift from 3448 cm^−1^ to 3418 cm^−1^ (purple dotted line) clearly convinced a successful amidation reaction between the acid group of KGN and the amino group of CS. As for the OSA polymer, the inert dihydroxy groups of alginates were feasibly oxidized to form the dialdehyde in the presence of oxidative NaIO_4_. As shown in Figure 2B, the generated two peaks at 5.3 ppm and 5.6 ppm in the ^1^H NMR spectra are ascribed to the hemiacetalic protons originating from the hydroxyl groups of aldehydes and their neighbors, demonstrating the preparation of the targeted OSA polymer. In this case, the CS-KGN/OSA composite hydrogel could be facilely yielded via the highly efficient Schiff base reaction between the aldehyde group of the OSA and amine groups of the CS-KGN composite in mild conditions. In addition to the chemical crosslinking, the physical interaction also played an important role in constructing the hydrogel network due to a few hydrophilic-group-induced electrostatic interactions, hydrogen–hydrogen interactions, and various molecular chain entanglements within these polymeric networks. Since the residual aldehyde group of the OSA polymer can continue to undergo a Schiff base reaction with the amine groups in the tissue protein once injected into the tissue defects, this hydrogel has the required strong tissue adhesion strength for application in osteochondral-related diseases.

### 3.2. The Porous Structures and Mechanical Properties of Hydrogels

**Figure 3:**
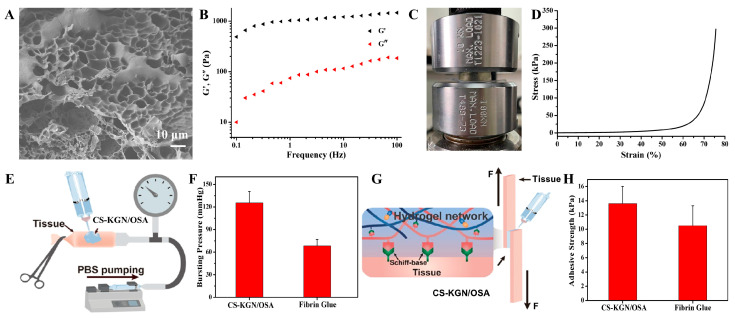
(**A**) SEM image, (**B**) rheological curve, and (**C**,**D**) compressive measurement of the CS-KGN/OSA hydrogel. (**E**,**F**) Schematic illustration of the bursting pressure measurement and comparative burst pressure of the CS-KGN/OSA hydrogel and fibrin glue. (**G**,**H**) Schematic illustration of the tissue adhesion using the lap shear measurement and comparative adhesion strength of the CS-KGN/OSA hydrogel and commercial fibrin glue on porcine skin.

The CS-KGN/OSA hydrogel was prepared via mixing the CS-KGN and OSA precursor solutions together with a dual-barrel syringe, presenting good gelation ability and easy injectability. The porous structure and pore size of the hydrogel scaffold is important for cell infiltration and nutrition transfer as well as other substance exchanges. For the composite CS-KGN/OSA hydrogel, Figure 3A shows the inner porous morphology, suitable size (ca. 20 μm), and porosities (75.04 ± 4.84%) that could satisfy the requirement of advantageous chondrogenic differentiation of BMSCs and nutrition and waste transfer for biological scaffolds. A variety of dense clusters in the SEM image were observed, verifying the complex polymeric interactions within the hydrogel networks. Moreover, these dense architectures also indicated the slowly sustained drug release from the network that may allow high drug concentration in situ over a longer period of time to promote cartilage tissue repair.

Furthermore, mechanical strength is another key aspect of hydrogel scaffolds because of the requirement of mechanical support for the implanted engineered scaffold. Before tissue regeneration, mechanical matching to the native cartilage in the defect area is beneficial in order to maintain scaffold stability and avoids random movements caused by exposure to external forces. Thus, rheological and compressive experiments were carried out and the results are shown in Figure 3B–D. The rheological curves in Figure 3B not only demonstrate the gel state with a higher storage modulus (G′) than loss modulus (G′′) within the whole frequency range but also indicate that the chemical grafting of KGN groups cannot alter the rigid backbones and mechanical properties of CS-based hydrogels. Figure 3C,D show that the CS-KGN/OSA hydrogel possesses robust mechanical properties with resilient elasticity and a compressive stress of 300 kPa at the strain of 80%, which may be ascribed to the rigid CS/SA backbones, dense network structures, and complex polymeric chain entanglements.

More importantly, on account of the residual aldehyde groups within the OSA backbone, the composite CS-KGN/OSA hydrogel also demonstrated attractive adhesive strengths in tissue applications owing to the generation of a number of Schiff base bonds between the aldehyde groups of the CS-KGN/OSA hydrogel and the amine groups of the tissue, which is important for clinical applications in order to dissipate energy through a flexible network and resist damage once exposed to external forces. Firstly, the burst pressure measurement was utilized to evaluate the adhesive strength and whether it could effectively seal the sustainable flow of fluid inside the artery. Figure 3E,F show that the bursting pressure of the CS-KGN/OSA hydrogel could achieve 123 mmHg, which is far beyond that of the clinically used fibrin glue. In addition, the adhesive strength of the hydrogel was also quantitatively assessed on the porcine tissue by a lap shear test in Figure 3G,H. Compared with the most commonly used control of fibrin glue, the CS-KGN/OSA hydrogel exhibited a higher interface adhesive strength of 13.8 kPa, which relied on the chemical reaction of the remaining aldehyde groups with the tissue amines; this result is indicative of a satisfactory adhesive property for the tissue-engineered scaffold.

### 3.3. The Swelling Property and Drug Release from the CS-KGN/OSA Hydrogel

**Figure 4:**
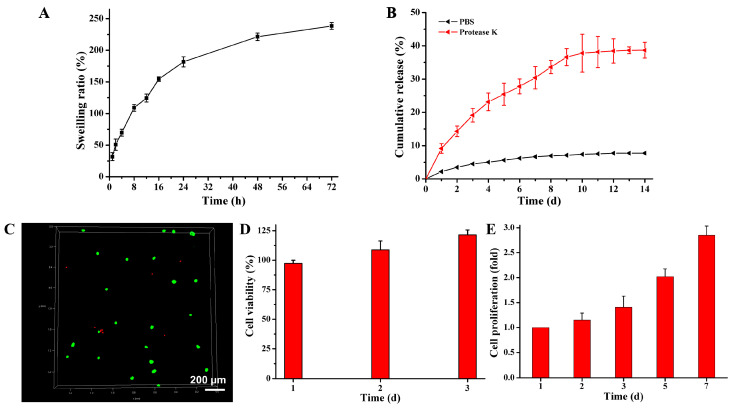
(**A**) The degradation rate of the composite CS-KGN/OSA hydrogel. (**B**) KGN release behavior from the composite CS-KGN/OSA hydrogel. (**C**) Live/dead staining of BMSCs after 3 days of culture in vitro. The green dots represent living cells and the red dots represent the dead cells. (**D**,**E**) Cell viability and proliferation of the CS-KGN/OSA hydrogel in vitro after cultivation at various time periods.

Figure 4A shows that the CS-KGN/OSA hydrogel could achieve a swelling equilibrium after 72 h of incubation in PBS solutions and exhibited a lower swelling ratio (238%) compared with traditional natural polysaccharide hydrogels, which was attributed to its physical chain entanglement and denser framework that could confine the water diffusion to the internal network to a certain degree. To the best of our knowledge, traditional regulatory factors (e.g., protein growth factors) possessed some fatal defects like short half-life, poor stability, and easy deactivation, which significantly limited the working activity and service life of the hydrogel within the implanted scaffolds for tissue regeneration. Thereafter, to address these troublesome issues, we used stable and non-toxic KGN agents as regulatory drugs to induce BMSCs into chondrocytes and promote cartilage regeneration. Compared with the physical encapsulation of KNG drugs, KGN moieties chemically linked into the network displayed slow-release behavior.

Proteinase K, also known as protease K or endopeptidase K, is a serine protease with wide cleavage activity. It can cut the carboxy-terminal peptide bonds of aliphatic and aromatic amino acids. Since protease K has the ability to degrade natural proteins, it has been widely used to digest various proteins in various molecular biology and cell biology methods and other applications, including preparation of chromosomal DNA by pulsed electrophoresis, Western blotting, removal of nucleases from DNA and RNA preparation, and enzyme digestion and removal, etc. The normal working concentration of protease K is 50–100 μg/mL, and it is active in a wide range of pH values (pH 4.0–12.5) [32,44]. Therefore, to simulate the degradation process in vivo, a PBS solution containing protease K was employed for scaffold degradation because the protease K could facilitate the degradation of the amide bond and the ester bond in solution. As shown in Figure 4B, compared with the PBS solution with only a 7.8% release rate, the incorporation of protease K in PBS solutions could indeed accelerate hydrogel degradation. In this case, the cumulative release achieved 38.7% with a slow, sustainable release behavior after 2 weeks, which could completely overcome the limitations of protein factor-loaded scaffolds and meet the requirement of long-lasting release with sufficient drug concentration in vivo (100 nM–100 μM) for immune regulation during a long period of time [33,45]. Moreover, it also indicated the suitable degradation of the implanted hydrogel scaffold to match the chondrogenic differentiation of BMSCs and promote cartilage repair.

### 3.4. Cell Biocompatibility of the CS-KGN/OSA Hydrogels

On account of the universal biocompatibility of naturally derived CS and SA polymers and non-protein chondrogenesis of the KGN molecule, the CS-KGN/OSA hydrogel should be favored as an effective hydrogel for its effects on cells and/or tissues. As shown in Figure 4C, the cytotoxicity assay showed that this biocompatible CS-KGN/OSA hydrogel could efficiently support cell growth and promote cell proliferation. After incubation for 3 days, an obviously increasing cell number with 123% cell viability fully demonstrated the excellent cytocompatibility of this hydrogel scaffold, which also indicated that the complete chemical grafting of KGN moieties onto the CS framework did not affect the material biosafety even though some of the KNG drugs may leak out of the CS-KGN/OSA hydrogel within 3 days. Actually, the seeded cells within the scaffolds could retain higher viability during the culturing period for more than a week. This advantageous cell growth and proliferation capacity reflects its potential as an alternative and ideal engineered scaffold in cartilage tissue engineering and regenerative medicine applications.

### 3.5. Chondrogenic Differentiation

**Figure 5:**
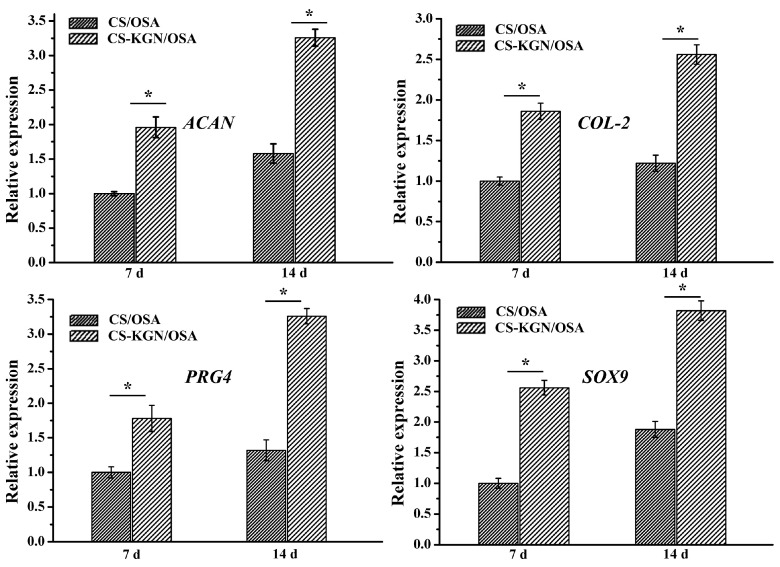
mRNA expression levels of cartilage-specific marker genes (*ACAN*, *COL2*, *PRG4,* and *SOX9*) for CS/OSA and CS-KGN/OSA scaffolds on days 7 and 14 (*n* = 3, * *p* < 0.05).

Taking into consideration the porous networks, favorable mechanics, and excellent biocompatibility of the composite hydrogel, this CS-KGN/OSA should be a good candidate for the chondrogenic differentiation of BMSCs and cartilage repair. Therefore, we investigated the chondrogenic differentiation by analyzing some important cartilage-specific marker gene expressions in vitro. After the chondrogenesis culture of BMSC with the hydrogel, Figure 5 shows the typical mRNA expression levels of cartilage-specific marker genes (*ACAN*, *COL2*, *PRG4*, and *SOX9*). To reveal the KGN effect on the chondrogenic differentiation, we prepared another CS/OSA hydrogel without KGN modification as a control. The real-time PCR results showed that all four cartilage-specific marker genes exhibited higher expression levels of the CS-KGN/OSA hydrogel than that of the CS/OSA hydrogel. In addition, the slow KGN release behavior endowed the CS-KGN/OSA hydrogel scaffold with beneficial and sustainable chondrogenesis because all the mRNA expression levels of cartilaginous markers were kept upregulated after incubation for 14 d, which suggested that the KGN drug could significantly induce the chondrogenesis of BMSCs for a long period of time in vitro. The non-KGN-laden CS/OSA hydrogel also exhibited upregulated gene expression from day 7 to day 14, which may be ascribed to its well-matched mechanics, adhesive strength, and excellent biocompatibility for promoting cell attachment, growth, proliferation, and differentiation in vitro.

**Figure 6:**
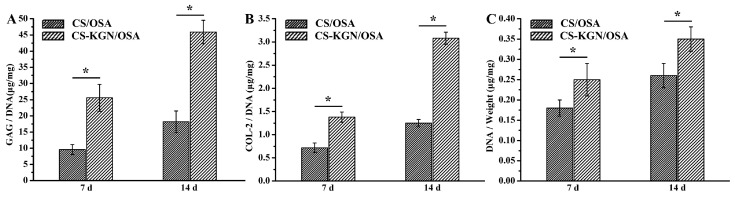
(**A**) GAG production, (**B**) COL2 proteins, and (**C**) DNA contents in hydrogel scaffolds of CS/OSA and CS-KGN/OSA after culturing for 7 and 14 d (*n* = 3, * *p* < 0.05).

The ELISA assay can be applied to quantitatively assess the extracellular protein levels in BMSCs within the biocompatible hydrogel scaffolds. Figure 6A shows that with the prolongation of time, the DNA content of CS-KGN/OSA and CS/OSA groups were increased during the two weeks. The higher DNA content of the CS-KGN/OSA hydrogel further indicated the effect of KGN in promoting chondrogenesis in vitro. Figure 6B,C show similar significant improvements relative to chondrogenic levels of GAG and COL-2, with higher levels of GAG and COL-2 on day 14 than in the CS/OSA group, respectively. These results powerfully disclose the synergistic effects of the biocompatible hydrogel and demonstrate its suitable mechanics, excellent cell proliferation, outstanding chondrogenic differentiation, and tailorable drug release in facilitating articular cartilage regeneration.

## 4. Conclusions

In summary, a novel KGN-conjugated composite hydrogel was fabricated with porous networks, easy injectability, suitable mechanics, favorable adhesion, low swelling ratio, and excellent compatibility via simple preparation approaches. In view of its good cell activity and proliferation capacities, this composite CS-KGN/OSA hydrogel is beneficial for maintaining cell viability, stable chondrogenic differentiation, and effective cartilage regeneration. In addition, on account of the sustainable KGN release from the chemically linked CS backbone for a long time, the typical cartilage-specific gene expressions were significantly upregulated while DNA level and GAG content were also increased after co-culturing with hydrogel and BMSCs in vitro, thus verifying the enduring chondrogenesis and great potential application prospects in a clinic setting. Therefore, this CS-KGN/OSA composite hydrogel not only furnishes surgeons with the availability of an injectable scaffold to fill in different gaps in cartilage defects, but also provides a hydrogel with stable chondrogenic activity to favor cartilage growth in the clinical treatment of bone-related diseases.

## Figures and Tables

**Table 1:** Primer sequences used for the RT-PCR.

Gene	Forward Primers (5′–3′)	Reverse Primers (5′–3′)
*COL2*	GCAGCTGTGTGCAGGAGGGGAAG	TGGCAGTGGCGAGGTCAGTAGGG
*ACAN*	GACTCATTGTTAGAGGACAGCCA	CACTCCCAAAAAGAACTCCAGAT
*PRG4*	GGCAGGGAATGTGACTGTGATG	TGGGTGAGCGTTTAGTTGTTGA
*SOX9*	CGGCGGAGGAAGTCGGTGAAGA	AGTGGTGGGTGGGGTGGTGGTG
*GAPDH*	CATCAAGAAGGTGGTGAAGCAGG	AGCATCGAAGGTAGAGGAGTGGG

*COL2*: type II collagen; *ACAN*: aggrecan; *PRG4*: proteoglycan 4 precursors; *SOX9*: SRY-related high mobility group-box gene 9; and *GAPDH*: glyceraldehyde-3-phosphate dehydrogenase.

## Data Availability

Data are contained within the article.
